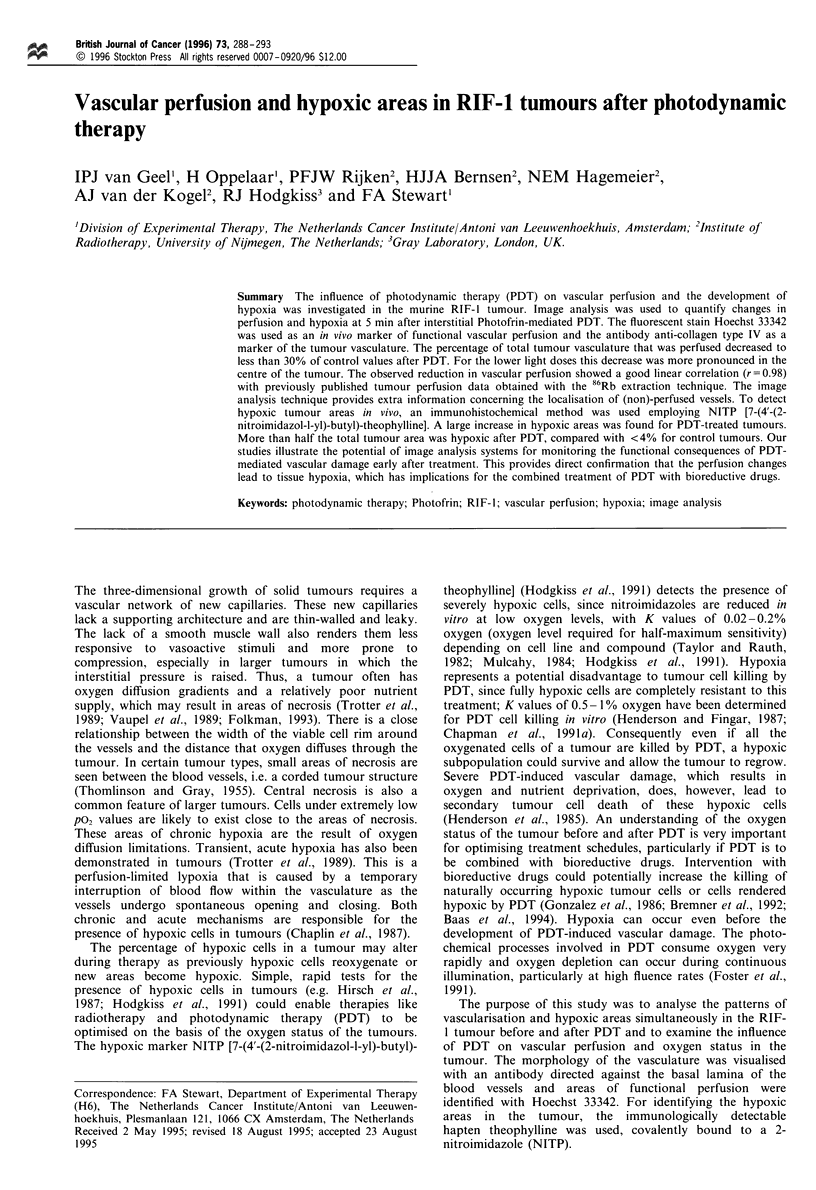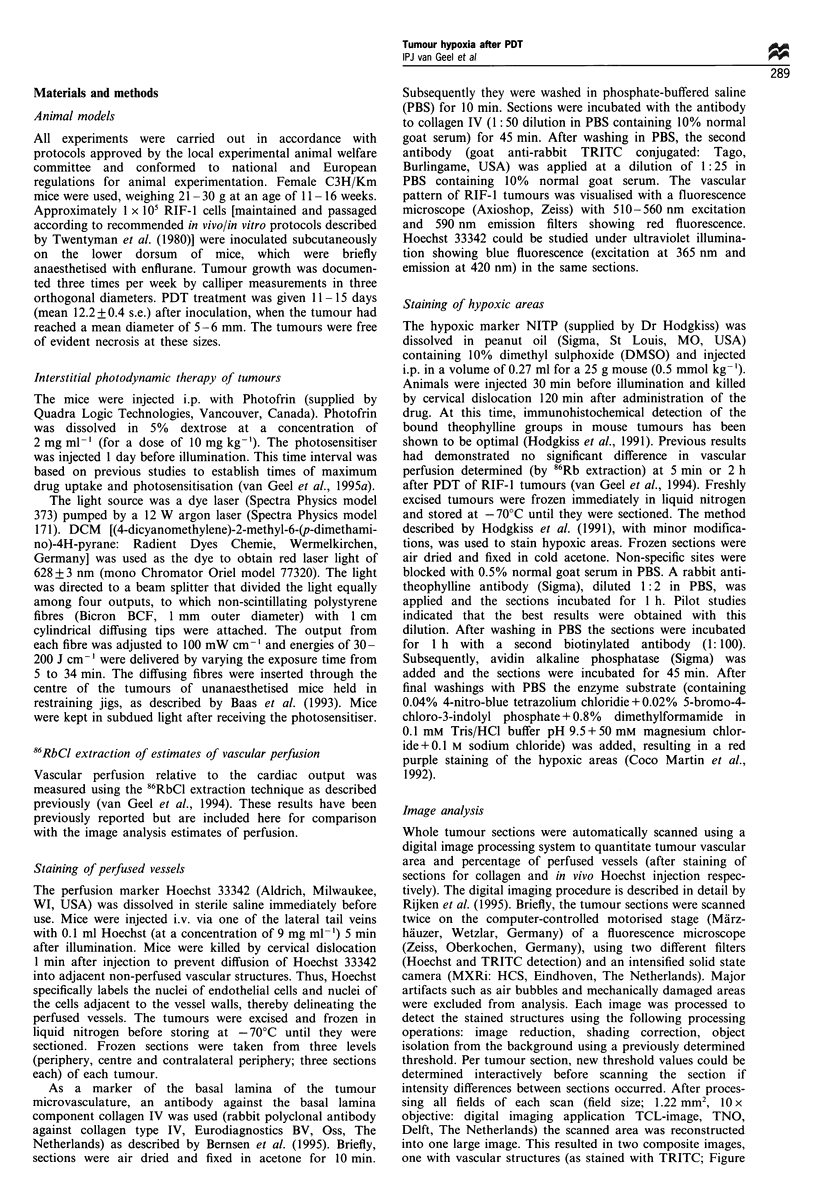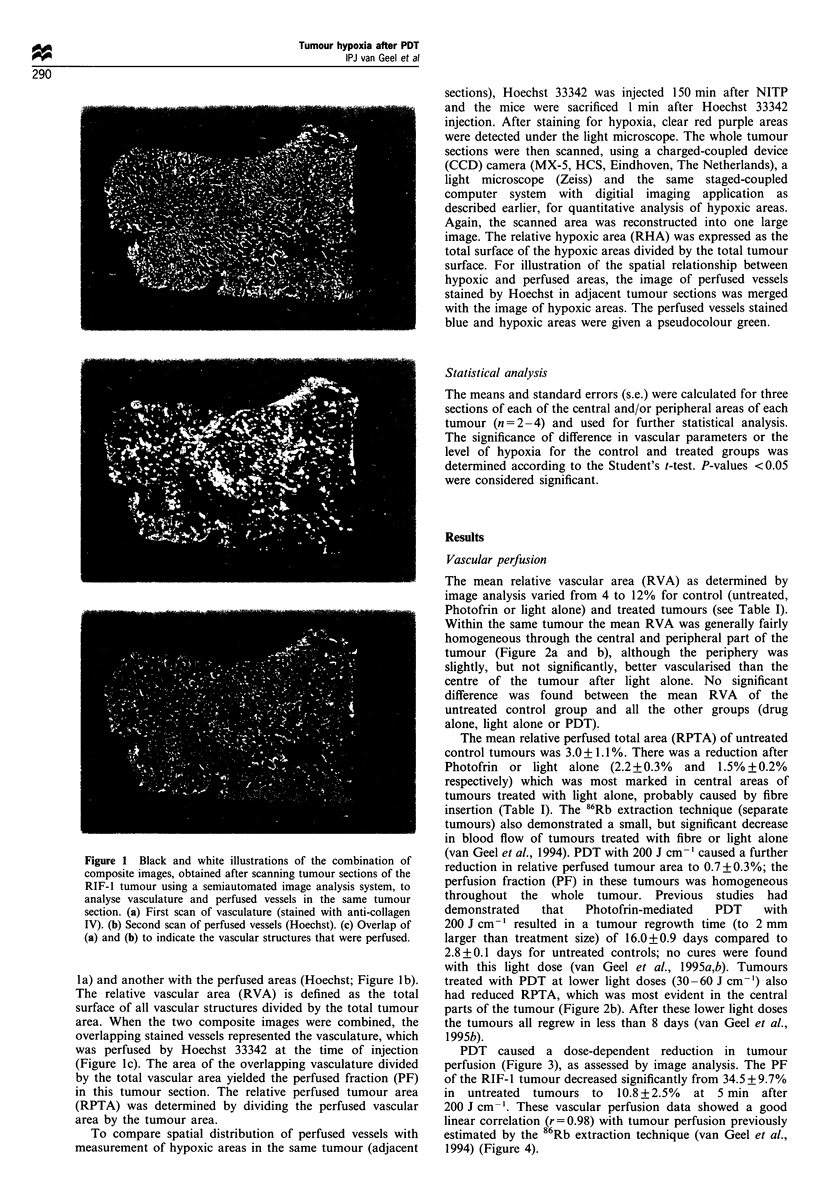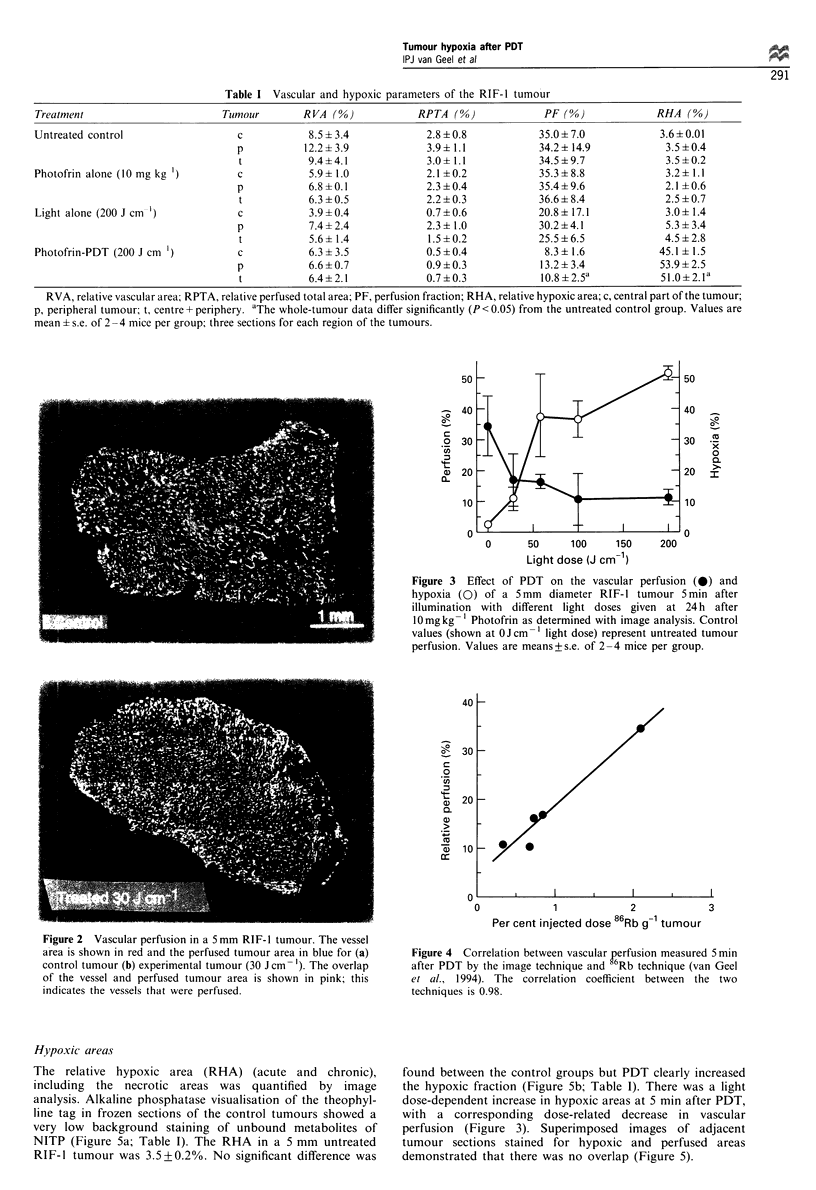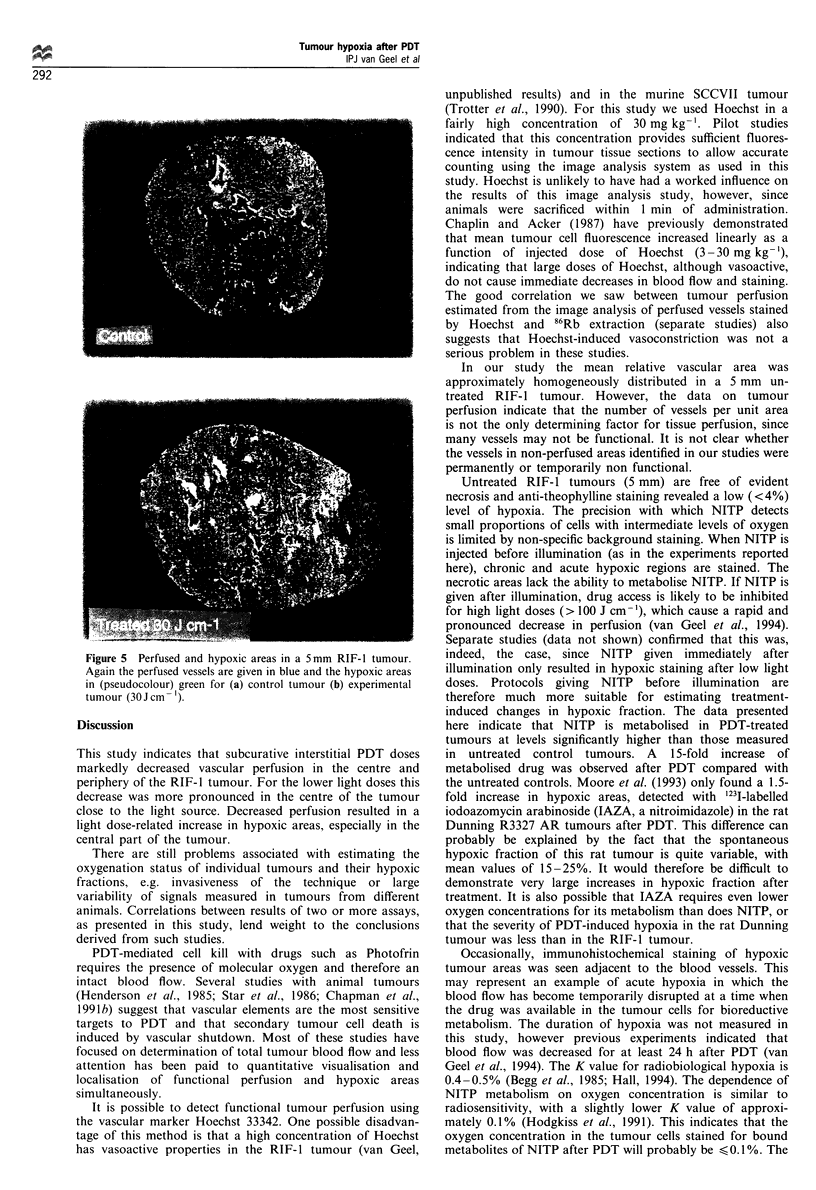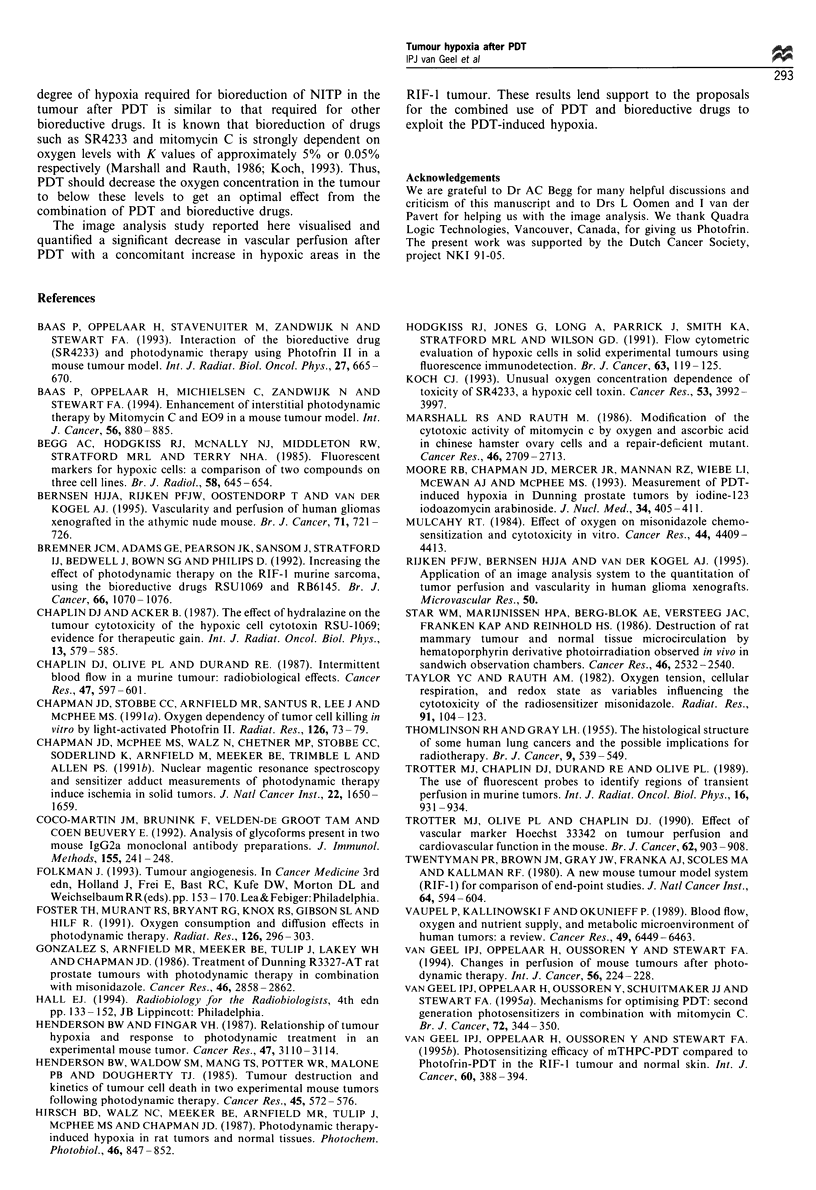# Vascular perfusion and hypoxic areas in RIF-1 tumours after photodynamic therapy.

**DOI:** 10.1038/bjc.1996.51

**Published:** 1996-02

**Authors:** I. P. van Geel, H. Oppelaar, P. F. Rijken, H. J. Bernsen, N. E. Hagemeier, A. J. van der Kogel, R. J. Hodgkiss, F. A. Stewart

**Affiliations:** Division of Experimental Therapy, Netherlands Cancer Institute/Antoni van Leeuwenhoekhuis, Amsterdam, The Netherlands.

## Abstract

**Images:**


British Journal of Cancer (1996) 73, 288-293
© 1996 Stockton Press All rights reserved 0007-0920/96 $12.00

### Vascular perfusion and hypoxic areas in RIF-1 tumours after photodynamic therapy

IPJ van Geel[1], H Oppelaar[1], PFJW Rijken[2], HJJA Bernsen[2], NEM Hagemeier[2], AJ van der Kogel[2], RJ Hodgkiss[3] and FA Stewart[1]

[1]*Division of Experimental Therapy, The Netherlands Cancer Institute/Antoni van Leeuwenhoekhuis, Amsterdam;* [2]*Institute of Radiotherapy, University of Nijmegen, The Netherlands;* [3]*Gray Laboratory, London, UK.*

**Summary** The influence of photodynamic therapy (PDT) on vascular perfusion and the development of hypoxia was investigated in the murine RIF-1 tumour. Image analysis was used to quantify changes in perfusion and hypoxia at 5 min after interstitial Photofrin-mediated PDT. The fluorescent stain Hoechst 33342 was used as an *in vivo* marker of functional vascular perfusion and the antibody anti-collagen type IV as a marker of the tumour vasculature. The percentage of total tumour vasculature that was perfused decreased to less than 30% of control values after PDT. For the lower light doses this decrease was more pronounced in the centre of the tumour. The observed reduction in vascular perfusion showed a good linear correlation ($r=0.98$) with previously published tumour perfusion data obtained with the $^{86}$Rb extraction technique. The image analysis technique provides extra information concerning the localisation of (non)-perfused vessels. To detect hypoxic tumour areas *in vivo*, an immunohistochemical method was used employing NITP [7-(4′-(2-nitroimidazol-l-yl)-butyl)-theophylline]. A large increase in hypoxic areas was found for PDT-treated tumours. More than half the total tumour area was hypoxic after PDT, compared with <4% for control tumours. Our studies illustrate the potential of image analysis systems for monitoring the functional consequences of PDT-mediated vascular damage early after treatment. This provides direct confirmation that the perfusion changes lead to tissue hypoxia, which has implications for the combined treatment of PDT with bioreductive drugs.

**Keywords:** photodynamic therapy; Photofrin; RIF-1; vascular perfusion; hypoxia; image analysis

The three-dimensional growth of solid tumours requires a vascular network of new capillaries. These new capillaries lack a supporting architecture and are thin-walled and leaky. The lack of a smooth muscle wall also renders them less responsive to vasoactive stimuli and more prone to compression, especially in larger tumours in which the interstitial pressure is raised. Thus, a tumour often has oxygen diffusion gradients and a relatively poor nutrient supply, which may result in areas of necrosis (Trotter *et al.*, 1989; Vaupel *et al.*, 1989; Folkman, 1993). There is a close relationship between the width of the viable cell rim around the vessels and the distance that oxygen diffuses through the tumour. In certain tumour types, small areas of necrosis are seen between the blood vessels, i.e. a corded tumour structure (Thomlinson and Gray, 1955). Central necrosis is also a common feature of larger tumours. Cells under extremely low $pO_2$ values are likely to exist close to the areas of necrosis. These areas of chronic hypoxia are the result of oxygen diffusion limitations. Transient, acute hypoxia has also been demonstrated in tumours (Trotter *et al.*, 1989). This is a perfusion-limited lypoxia that is caused by a temporary interruption of blood flow within the vasculature as the vessels undergo spontaneous opening and closing. Both chronic and acute mechanisms are responsible for the presence of hypoxic cells in tumours (Chaplin *et al.*, 1987).

The percentage of hypoxic cells in a tumour may alter during therapy as previously hypoxic cells reoxygenate or new areas become hypoxic. Simple, rapid tests for the presence of hypoxic cells in tumours (e.g. Hirsch *et al.*, 1987; Hodgkiss *et al.*, 1991) could enable therapies like radiotherapy and photodynamic therapy (PDT) to be optimised on the basis of the oxygen status of the tumours. The hypoxic marker NITP [7-(4′-(2-nitroimidazol-l-yl)-butyl)-theophylline] (Hodgkiss *et al.*, 1991) detects the presence of severely hypoxic cells, since nitroimidazoles are reduced *in vitro* at low oxygen levels, with $K$ values of 0.02–0.2% oxygen (oxygen level required for half-maximum sensitivity) depending on cell line and compound (Taylor and Rauth, 1982; Mulcahy, 1984; Hodgkiss *et al.*, 1991). Hypoxia represents a potential disadvantage to tumour cell killing by PDT, since fully hypoxic cells are completely resistant to this treatment; $K$ values of 0.5–1% oxygen have been determined for PDT cell killing *in vitro* (Henderson and Fingar, 1987; Chapman *et al.*, 1991*a*). Consequently even if all the oxygenated cells of a tumour are killed by PDT, a hypoxic subpopulation could survive and allow the tumour to regrow. Severe PDT-induced vascular damage, which results in oxygen and nutrient deprivation, does, however, lead to secondary tumour cell death of these hypoxic cells (Henderson *et al.*, 1985). An understanding of the oxygen status of the tumour before and after PDT is very important for optimising treatment schedules, particularly if PDT is to be combined with bioreductive drugs. Intervention with bioreductive drugs could potentially increase the killing of naturally occurring hypoxic tumour cells or cells rendered hypoxic by PDT (Gonzalez *et al.*, 1986; Bremner *et al.*, 1992; Baas *et al.*, 1994). Hypoxia can occur even before the development of PDT-induced vascular damage. The photochemical processes involved in PDT consume oxygen very rapidly and oxygen depletion can occur during continuous illumination, particularly at high fluence rates (Foster *et al.*, 1991).

The purpose of this study was to analyse the patterns of vascularisation and hypoxic areas simultaneously in the RIF-1 tumour before and after PDT and to examine the influence of PDT on vascular perfusion and oxygen status in the tumour. The morphology of the vasculature was visualised with an antibody directed against the basal lamina of the blood vessels and areas of functional perfusion were identified with Hoechst 33342. For identifying the hypoxic areas in the tumour, the immunologically detectable hapten theophylline was used, covalently bound to a 2-nitroimidazole (NITP).

Correspondence: FA Stewart, Department of Experimental Therapy (H6), The Netherlands Cancer Institute/Antoni van Leeuwenhoekhuis, Plesmanlaan 121, 1066 CX Amsterdam, The Netherlands
Received 2 May 1995; revised 18 August 1995; accepted 23 August 1995

### Materials and methods

### *Animal models*

All experiments were carried out in accordance with protocols approved by the local experimental animal welfare committee and conformed to national and European regulations for animal experimentation. Female C3H/Km mice were used, weighing 21–30 g at an age of 11–16 weeks. Approximately $1 \times 10^5$ RIF-1 cells [maintained and passaged according to recommended *in vivo/in vitro* protocols described by Twentyman *et al.* (1980)] were inoculated subcutaneously on the lower dorsum of mice, which were briefly anaesthetised with enflurane. Tumour growth was documented three times per week by calliper measurements in three orthogonal diameters. PDT treatment was given 11–15 days (mean $12.2 \pm 0.4$ s.e.) after inoculation, when the tumour had reached a mean diameter of 5–6 mm. The tumours were free of evident necrosis at these sizes.

### *Interstitial photodynamic therapy of tumours*

The mice were injected i.p. with Photofrin (supplied by Quadra Logic Technologies, Vancouver, Canada). Photofrin was dissolved in 5% dextrose at a concentration of 2 mg $ml^{-1}$ (for a dose of 10 mg $kg^{-1}$). The photosensitiser was injected 1 day before illumination. This time interval was based on previous studies to establish times of maximum drug uptake and photosensitisation (van Geel *et al.*, 1995*a*).

The light source was a dye laser (Spectra Physics model 373) pumped by a 12 W argon laser (Spectra Physics model 171). DCM [(4-dicyanomethylene)-2-methyl-6-(*p*-dimethamino)-4H-pyrane: Radient Dyes Chemie, Wermelkirchen, Germany] was used as the dye to obtain red laser light of $628 \pm 3$ nm (mono Chromator Oriel model 77320). The light was directed to a beam splitter that divided the light equally among four outputs, to which non-scintillating polystyrene fibres (Bicron BCF, 1 mm outer diameter) with 1 cm cylindrical diffusing tips were attached. The output from each fibre was adjusted to 100 mW $cm^{-1}$ and energies of 30–200 J $cm^{-1}$ were delivered by varying the exposure time from 5 to 34 min. The diffusing fibres were inserted through the centre of the tumours of unanaesthetised mice held in restraining jigs, as described by Baas *et al.* (1993). Mice were kept in subdued light after receiving the photosensitiser.

### *$^{86}RbCl$ extraction of estimates of vascular perfusion*

Vascular perfusion relative to the cardiac output was measured using the $^{86}RbCl$ extraction technique as described previously (van Geel *et al.*, 1994). These results have been previously reported but are included here for comparison with the image analysis estimates of perfusion.

### *Staining of perfused vessels*

The perfusion marker Hoechst 33342 (Aldrich, Milwaukee, WI, USA) was dissolved in sterile saline immediately before use. Mice were injected i.v. via one of the lateral tail veins with 0.1 ml Hoechst (at a concentration of 9 mg $ml^{-1}$) 5 min after illumination. Mice were killed by cervical dislocation 1 min after injection to prevent diffusion of Hoechst 33342 into adjacent non-perfused vascular structures. Thus, Hoechst specifically labels the nuclei of endothelial cells and nuclei of the cells adjacent to the vessel walls, thereby delineating the perfused vessels. The tumours were excised and frozen in liquid nitrogen before storing at −70°C until they were sectioned. Frozen sections were taken from three levels (periphery, centre and contralateral periphery; three sections each) of each tumour.

As a marker of the basal lamina of the tumour microvasculature, an antibody against the basal lamina component collagen IV was used (rabbit polyclonal antibody against collagen type IV, Eurodiagnostics BV, Oss, The Netherlands) as described by Bernsen *et al.* (1995). Briefly, sections were air dried and fixed in acetone for 10 min. Subsequently they were washed in phosphate-buffered saline (PBS) for 10 min. Sections were incubated with the antibody to collagen IV (1:50 dilution in PBS containing 10% normal goat serum) for 45 min. After washing in PBS, the second antibody (goat anti-rabbit TRITC conjugated: Tago, Burlingame, USA) was applied at a dilution of 1:25 in PBS containing 10% normal goat serum. The vascular pattern of RIF-1 tumours was visualised with a fluorescence microscope (Axioshop, Zeiss) with 510–560 nm excitation and 590 nm emission filters showing red fluorescence. Hoechst 33342 could be studied under ultraviolet illumination showing blue fluorescence (excitation at 365 nm and emission at 420 nm) in the same sections.

### *Staining of hypoxic areas*

The hypoxic marker NITP (supplied by Dr Hodgkiss) was dissolved in peanut oil (Sigma, St Louis, MO, USA) containing 10% dimethyl sulphoxide (DMSO) and injected i.p. in a volume of 0.27 ml for a 25 g mouse (0.5 mmol $kg^{-1}$). Animals were injected 30 min before illumination and killed by cervical dislocation 120 min after administration of the drug. At this time, immunohistochemical detection of the bound theophylline groups in mouse tumours has been shown to be optimal (Hodgkiss *et al.*, 1991). Previous results had demonstrated no significant difference in vascular perfusion determined (by $^{86}Rb$ extraction) at 5 min or 2 h after PDT of RIF-1 tumours (van Geel *et al.*, 1994). Freshly excised tumours were frozen immediately in liquid nitrogen and stored at −70°C until they were sectioned. The method described by Hodgkiss *et al.* (1991), with minor modifications, was used to stain hypoxic areas. Frozen sections were air dried and fixed in cold acetone. Non-specific sites were blocked with 0.5% normal goat serum in PBS. A rabbit anti-theophylline antibody (Sigma), diluted 1:2 in PBS, was applied and the sections incubated for 1 h. Pilot studies indicated that the best results were obtained with this dilution. After washing in PBS the sections were incubated for 1 h with a second biotinylated antibody (1:100). Subsequently, avidin alkaline phosphatase (Sigma) was added and the sections were incubated for 45 min. After final washings with PBS the enzyme substrate (containing 0.04% 4-nitro-blue tetrazolium chloridie + 0.02% 5-bromo-4-chloro-3-indolyl phosphate + 0.8% dimethylformamide in 0.1 mM Tris/HCl buffer pH 9.5 + 50 mM magnesium chloride + 0.1 M sodium chloride) was added, resulting in a red purple staining of the hypoxic areas (Coco Martin *et al.*, 1992).

### *Image analysis*

Whole tumour sections were automatically scanned using a digital image processing system to quantitate tumour vascular area and percentage of perfused vessels (after staining of sections for collagen and *in vivo* Hoechst injection respectively). The digital imaging procedure is described in detail by Rijken *et al.* (1995). Briefly, the tumour sections were scanned twice on the computer-controlled motorised stage (Märzhäuzer, Wetzlar, Germany) of a fluorescence microscope (Zeiss, Oberkochen, Germany), using two different filters (Hoechst and TRITC detection) and an intensified solid state camera (MXRi: HCS, Eindhoven, The Netherlands). Major artifacts such as air bubbles and mechanically damaged areas were excluded from analysis. Each image was processed to detect the stained structures using the following processing operations: image reduction, shading correction, object isolation from the background using a previously determined threshold. Per tumour section, new threshold values could be determined interactively before scanning the section if intensity differences between sections occurred. After processing all fields of each scan (field size; 1.22 $mm^2$, 10× objective: digital imaging application TCL-image, TNO, Delft, The Netherlands) the scanned area was reconstructed into one large image. This resulted in two composite images, one with vascular structures (as stained with TRITC; Figure

**Figure 1** Black and white illustrations of the combination of composite images, obtained after scanning tumour sections of the RIF-1 tumour using a semiautomated image analysis system, to analyse vasculature and perfused vessels in the same tumour section. (**a**) First scan of vasculature (stained with anti-collagen IV). (**b**) Second scan of perfused vessels (Hoechst). (**c**) Overlap of (**a**) and (**b**) to indicate the vascular structures that were perfused.

1a) and another with the perfused areas (Hoechst; Figure 1b). The relative vascular area (RVA) is defined as the total surface of all vascular structures divided by the total tumour area. When the two composite images were combined, the overlapping stained vessels represented the vasculature, which was perfused by Hoechst 33342 at the time of injection (Figure 1c). The area of the overlapping vasculature divided by the total vascular area yielded the perfused fraction (PF) in this tumour section. The relative perfused tumour area (RPTA) was determined by dividing the perfused vascular area by the tumour area.

To compare spatial distribution of perfused vessels with measurement of hypoxic areas in the same tumour (adjacent sections), Hoechst 33342 was injected 150 min after NITP and the mice were sacrificed 1 min after Hoechst 33342 injection. After staining for hypoxia, clear red purple areas were detected under the light microscope. The whole tumour sections were then scanned, using a charged-coupled device (CCD) camera (MX-5, HCS, Eindhoven, The Netherlands), a light microscope (Zeiss) and the same staged-coupled computer system with digitial imaging application as described earlier, for quantitative analysis of hypoxic areas. Again, the scanned area was reconstructed into one large image. The relative hypoxic area (RHA) was expressed as the total surface of the hypoxic areas divided by the total tumour surface. For illustration of the spatial relationship between hypoxic and perfused areas, the image of perfused vessels stained by Hoechst in adjacent tumour sections was merged with the image of hypoxic areas. The perfused vessels stained blue and hypoxic areas were given a pseudocolour green.

*Statistical analysis*

The means and standard errors (s.e.) were calculated for three sections of each of the central and/or peripheral areas of each tumour ($n=2-4$) and used for further statistical analysis. The significance of difference in vascular parameters or the level of hypoxia for the control and treated groups was determined according to the Student's $t$-test. $P$-values $<0.05$ were considered significant.

### Results

*Vascular perfusion*

The mean relative vascular area (RVA) as determined by image analysis varied from 4 to 12% for control (untreated, Photofrin or light alone) and treated tumours (see Table I). Within the same tumour the mean RVA was generally fairly homogeneous through the central and peripheral part of the tumour (Figure 2a and b), although the periphery was slightly, but not significantly, better vascularised than the centre of the tumour after light alone. No significant difference was found between the mean RVA of the untreated control group and all the other groups (drug alone, light alone or PDT).

The mean relative perfused total area (RPTA) of untreated control tumours was $3.0 \pm 1.1\%$. There was a reduction after Photofrin or light alone ($2.2 \pm 0.3\%$ and $1.5\% \pm 0.2\%$ respectively) which was most marked in central areas of tumours treated with light alone, probably caused by fibre insertion (Table I). The $^{86}$Rb extraction technique (separate tumours) also demonstrated a small, but significant decrease in blood flow of tumours treated with fibre or light alone (van Geel *et al.*, 1994). PDT with 200 J cm$^{-1}$ caused a further reduction in relative perfused tumour area to $0.7 \pm 0.3\%$; the perfusion fraction (PF) in these tumours was homogeneous throughout the whole tumour. Previous studies had demonstrated that Photofrin-mediated PDT with 200 J cm$^{-1}$ resulted in a tumour regrowth time (to 2 mm larger than treatment size) of $16.0 \pm 0.9$ days compared to $2.8 \pm 0.1$ days for untreated controls; no cures were found with this light dose (van Geel *et al.*, 1995*a*,*b*). Tumours treated with PDT at lower light doses (30–60 J cm$^{-1}$) also had reduced RPTA, which was most evident in the central parts of the tumour (Figure 2b). After these lower light doses the tumours all regrew in less than 8 days (van Geel *et al.*, 1995*b*).

PDT caused a dose-dependent reduction in tumour perfusion (Figure 3), as assessed by image analysis. The PF of the RIF-1 tumour decreased significantly from $34.5 \pm 9.7\%$ in untreated tumours to $10.8 \pm 2.5\%$ at 5 min after 200 J cm$^{-1}$. These vascular perfusion data showed a good linear correlation ($r=0.98$) with tumour perfusion previously estimated by the $^{86}$Rb extraction technique (van Geel *et al.*, 1994) (Figure 4).

**Table I** Vascular and hypoxic parameters of the RIF-1 tumour

| *Treatment* | *Tumour* | *RVA (%)* | *RPTA (%)* | *PF (%)* | *RHA (%)* |
|---|---|---|---|---|---|
| Untreated control | c | 8.5 ± 3.4 | 2.8 ± 0.8 | 35.0 ± 7.0 | 3.6 ± 0.01 |
| | p | 12.2 ± 3.9 | 3.9 ± 1.1 | 34.2 ± 14.9 | 3.5 ± 0.4 |
| | t | 9.4 ± 4.1 | 3.0 ± 1.1 | 34.5 ± 9.7 | 3.5 ± 0.2 |
| Photofrin alone (10 mg kg$^{-1}$) | c | 5.9 ± 1.0 | 2.1 ± 0.2 | 35.3 ± 8.8 | 3.2 ± 1.1 |
| | p | 6.8 ± 0.1 | 2.3 ± 0.4 | 35.4 ± 9.6 | 2.1 ± 0.6 |
| | t | 6.3 ± 0.5 | 2.2 ± 0.3 | 36.6 ± 8.4 | 2.5 ± 0.7 |
| Light alone (200 J cm$^{-1}$) | c | 3.9 ± 0.4 | 0.7 ± 0.6 | 20.8 ± 17.1 | 3.0 ± 1.4 |
| | p | 7.4 ± 2.4 | 2.3 ± 1.0 | 30.2 ± 4.1 | 5.3 ± 3.4 |
| | t | 5.6 ± 1.4 | 1.5 ± 0.2 | 25.5 ± 6.5 | 4.5 ± 2.8 |
| Photofrin-PDT (200 J cm$^{-1}$) | c | 6.3 ± 3.5 | 0.5 ± 0.4 | 8.3 ± 1.6 | 45.1 ± 1.5 |
| | p | 6.6 ± 0.7 | 0.9 ± 0.3 | 13.2 ± 3.4 | 53.9 ± 2.5 |
| | t | 6.4 ± 2.1 | 0.7 ± 0.3 | 10.8 ± 2.5[a] | 51.0 ± 2.1[a] |

RVA, relative vascular area; RPTA, relative perfused total area; PF, perfusion fraction; RHA, relative hypoxic area; c, central part of the tumour; p, peripheral tumour; t, centre + periphery. [a]The whole-tumour data differ significantly ($P < 0.05$) from the untreated control group. Values are mean ± s.e. of 2–4 mice per group; three sections for each region of the tumours.

**Figure 2** Vascular perfusion in a 5 mm RIF-1 tumour. The vessel area is shown in red and the perfused tumour area in blue for (**a**) control tumour (**b**) experimental tumour (30 J cm$^{-1}$). The overlap of the vessel and perfused tumour area is shown in pink; this indicates the vessels that were perfused.

**Figure 3** Effect of PDT on the vascular perfusion (●) and hypoxia (○) of a 5 mm diameter RIF-1 tumour 5 min after illumination with different light doses given at 24 h after 10 mg kg$^{-1}$ Photofrin as determined with image analysis. Control values (shown at 0 J cm$^{-1}$ light dose) represent untreated tumour perfusion. Values are means ± s.e. of 2–4 mice per group.

**Figure 4** Correlation between vascular perfusion measured 5 min after PDT by the image technique and $^{86}Rb$ technique (van Geel *et al.*, 1994). The correlation coefficient between the two techniques is 0.98.

*Hypoxic areas*

The relative hypoxic area (RHA) (acute and chronic), including the necrotic areas was quantified by image analysis. Alkaline phosphatase visualisation of the theophylline tag in frozen sections of the control tumours showed a very low background staining of unbound metabolites of NITP (Figure 5a; Table I). The RHA in a 5 mm untreated RIF-1 tumour was 3.5 ± 0.2%. No significant difference was found between the control groups but PDT clearly increased the hypoxic fraction (Figure 5b; Table I). There was a light dose-dependent increase in hypoxic areas at 5 min after PDT, with a corresponding dose-related decrease in vascular perfusion (Figure 3). Superimposed images of adjacent tumour sections stained for hypoxic and perfused areas demonstrated that there was no overlap (Figure 5).

**Figure 5** Perfused and hypoxic areas in a 5 mm RIF-1 tumour. Again the perfused vessels are given in blue and the hypoxic areas in (pseudocolour) green for (**a**) control tumour (**b**) experimental tumour (30 J $cm^{-1}$).

### Discussion

This study indicates that subcurative interstitial PDT doses markedly decreased vascular perfusion in the centre and periphery of the RIF-1 tumour. For the lower light doses this decrease was more pronounced in the centre of the tumour close to the light source. Decreased perfusion resulted in a light dose-related increase in hypoxic areas, especially in the central part of the tumour.

There are still problems associated with estimating the oxygenation status of individual tumours and their hypoxic fractions, e.g. invasiveness of the technique or large variability of signals measured in tumours from different animals. Correlations between results of two or more assays, as presented in this study, lend weight to the conclusions derived from such studies.

PDT-mediated cell kill with drugs such as Photofrin requires the presence of molecular oxygen and therefore an intact blood flow. Several studies with animal tumours (Henderson *et al.*, 1985; Star *et al.*, 1986; Chapman *et al.*, 1991*b*) suggest that vascular elements are the most sensitive targets to PDT and that secondary tumour cell death is induced by vascular shutdown. Most of these studies have focused on determination of total tumour blood flow and less attention has been paid to quantitative visualisation and localisation of functional perfusion and hypoxic areas simultaneously.

It is possible to detect functional tumour perfusion using the vascular marker Hoechst 33342. One possible disadvantage of this method is that a high concentration of Hoechst has vasoactive properties in the RIF-1 tumour (van Geel, unpublished results) and in the murine SCCVII tumour (Trotter *et al.*, 1990). For this study we used Hoechst in a fairly high concentration of 30 mg $kg^{-1}$. Pilot studies indicated that this concentration provides sufficient fluorescence intensity in tumour tissue sections to allow accurate counting using the image analysis system as used in this study. Hoechst is unlikely to have had a worked influence on the results of this image analysis study, however, since animals were sacrificed within 1 min of administration. Chaplin and Acker (1987) have previously demonstrated that mean tumour cell fluorescence increased linearly as a function of injected dose of Hoechst (3–30 mg $kg^{-1}$), indicating that large doses of Hoechst, although vasoactive, do not cause immediate decreases in blood flow and staining. The good correlation we saw between tumour perfusion estimated from the image analysis of perfused vessels stained by Hoechst and $^{86}Rb$ extraction (separate studies) also suggests that Hoechst-induced vasoconstriction was not a serious problem in these studies.

In our study the mean relative vascular area was approximately homogeneously distributed in a 5 mm untreated RIF-1 tumour. However, the data on tumour perfusion indicate that the number of vessels per unit area is not the only determining factor for tissue perfusion, since many vessels may not be functional. It is not clear whether the vessels in non-perfused areas identified in our studies were permanently or temporarily non functional.

Untreated RIF-1 tumours (5 mm) are free of evident necrosis and anti-theophylline staining revealed a low (<4%) level of hypoxia. The precision with which NITP detects small proportions of cells with intermediate levels of oxygen is limited by non-specific background staining. When NITP is injected before illumination (as in the experiments reported here), chronic and acute hypoxic regions are stained. The necrotic areas lack the ability to metabolise NITP. If NITP is given after illumination, drug access is likely to be inhibited for high light doses (>100 J $cm^{-1}$), which cause a rapid and pronounced decrease in perfusion (van Geel *et al.*, 1994). Separate studies (data not shown) confirmed that this was, indeed, the case, since NITP given immediately after illumination only resulted in hypoxic staining after low light doses. Protocols giving NITP before illumination are therefore much more suitable for estimating treatment-induced changes in hypoxic fraction. The data presented here indicate that NITP is metabolised in PDT-treated tumours at levels significantly higher than those measured in untreated control tumours. A 15-fold increase of metabolised drug was observed after PDT compared with the untreated controls. Moore *et al.* (1993) only found a 1.5-fold increase in hypoxic areas, detected with $^{123}I$-labelled iodoazomycin arabinoside (IAZA, a nitroimidazole) in the rat Dunning R3327 AR tumours after PDT. This difference can probably be explained by the fact that the spontaneous hypoxic fraction of this rat tumour is quite variable, with mean values of 15–25%. It would therefore be difficult to demonstrate very large increases in hypoxic fraction after treatment. It is also possible that IAZA requires even lower oxygen concentrations for its metabolism than does NITP, or that the severity of PDT-induced hypoxia in the rat Dunning tumour was less than in the RIF-1 tumour.

Occasionally, immunohistochemical staining of hypoxic tumour areas was seen adjacent to the blood vessels. This may represent an example of acute hypoxia in which the blood flow has become temporarily disrupted at a time when the drug was available in the tumour cells for bioreductive metabolism. The duration of hypoxia was not measured in this study, however previous experiments indicated that blood flow was decreased for at least 24 h after PDT (van Geel *et al.*, 1994). The $K$ value for radiobiological hypoxia is 0.4–0.5% (Begg *et al.*, 1985; Hall, 1994). The dependence of NITP metabolism on oxygen concentration is similar to radiosensitivity, with a slightly lower $K$ value of approximately 0.1% (Hodgkiss *et al.*, 1991). This indicates that the oxygen concentration in the tumour cells stained for bound metabolites of NITP after PDT will probably be $\leqslant$0.1%. The degree of hypoxia required for bioreduction of NITP in the tumour after PDT is similar to that required for other bioreductive drugs. It is known that bioreduction of drugs such as SR4233 and mitomycin C is strongly dependent on oxygen levels with *K* values of approximately 5% or 0.05% respectively (Marshall and Rauth, 1986; Koch, 1993). Thus, PDT should decrease the oxygen concentration in the tumour to below these levels to get an optimal effect from the combination of PDT and bioreductive drugs.

The image analysis study reported here visualised and quantified a significant decrease in vascular perfusion after PDT with a concomitant increase in hypoxic areas in the RIF-1 tumour. These results lend support to the proposals for the combined use of PDT and bioreductive drugs to exploit the PDT-induced hypoxia.

**Acknowledgements**
We are grateful to Dr AC Begg for many helpful discussions and criticism of this manuscript and to Drs L Oomen and I van der Pavert for helping us with the image analysis. We thank Quadra Logic Technologies, Vancouver, Canada, for giving us Photofrin. The present work was supported by the Dutch Cancer Society, project NKI 91-05.

### References

BAAS P, OPPELAAR H, STAVENUITER M, ZANDWIJK N AND STEWART FA. (1993). Interaction of the bioreductive drug (SR4233) and photodynamic therapy using Photofrin II in a mouse tumour model. *Int. J. Radiat. Biol. Oncol. Phys.*, **27,** 665–670.

BAAS P, OPPELAAR H, MICHIELSEN C, ZANDWIJK N AND STEWART FA. (1994). Enhancement of interstitial photodynamic therapy by Mitomycin C and EO9 in a mouse tumour model. *Int. J. Cancer*, **56,** 880–885.

BEGG AC, HODGKISS RJ, MCNALLY NJ, MIDDLETON RW, STRATFORD MRL AND TERRY NHA. (1985). Fluorescent markers for hypoxic cells: a comparison of two compounds on three cell lines. *Br. J. Radiol.*, **58,** 645–654.

BERNSEN HJJA, RIJKEN PFJW, OOSTENDORP T AND VAN DER KOGEL AJ. (1995). Vascularity and perfusion of human gliomas xenografted in the athymic nude mouse. *Br. J. Cancer*, **71,** 721–726.

BREMNER JCM, ADAMS GE, PEARSON JK, SANSOM J, STRATFORD IJ, BEDWELL J, BOWN SG AND PHILIPS D. (1992). Increasing the effect of photodynamic therapy on the RIF-1 murine sarcoma, using the bioreductive drugs RSU1069 and RB6145. *Br. J. Cancer*, **66,** 1070–1076.

CHAPLIN DJ AND ACKER B. (1987). The effect of hydralazine on the tumour cytotoxicity of the hypoxic cell cytotoxin RSU-1069; evidence for therapeutic gain. *Int. J. Radiat. Oncol. Biol. Phys.*, **13,** 579–585.

CHAPLIN DJ, OLIVE PL AND DURAND RE. (1987). Intermittent blood flow in a murine tumour: radiobiological effects. *Cancer Res.*, **47,** 597–601.

CHAPMAN JD, STOBBE CC, ARNFIELD MR, SANTUS R, LEE J AND MCPHEE MS. (1991*a*). Oxygen dependency of tumor cell killing *in vitro* by light-activated Photofrin II. *Radiat. Res.*, **126,** 73–79.

CHAPMAN JD, MCPHEE MS, WALZ N, CHETNER MP, STOBBE CC, SODERLIND K, ARNFIELD M, MEEKER BE, TRIMBLE L AND ALLEN PS. (1991*b*). Nuclear magentic resonance spectroscopy and sensitizer adduct measurements of photodynamic therapy induce ischemia in solid tumors. *J. Natl Cancer Inst.*, **22,** 1650–1659.

COCO-MARTIN JM, BRUNINK F, VELDEN-DE GROOT TAM AND COEN BEUVERY E. (1992). Analysis of glycoforms present in two mouse IgG2a monoclonal antibody preparations. *J. Immunol. Methods*, **155,** 241–248.

FOLKMAN J. (1993). Tumour angiogenesis. In *Cancer Medicine* 3rd edn, Holland J, Frei E, Bast RC, Kufe DW, Morton DL and Weichselbaum RR (eds). pp. 153–170. Lea & Febiger: Philadelphia.

FOSTER TH, MURANT RS, BRYANT RG, KNOX RS, GIBSON SL AND HILF R. (1991). Oxygen consumption and diffusion effects in photodynamic therapy. *Radiat. Res.*, **126,** 296–303.

GONZALEZ S, ARNFIELD MR, MEEKER BE, TULIP J, LAKEY WH AND CHAPMAN JD. (1986). Treatment of Dunning R3327-AT rat prostate tumours with photodynamic therapy in combination with misonidazole. *Cancer Res.*, **46,** 2858–2862.

HALL EJ. (1994). *Radiobiology for the Radiobiologists*, 4th edn pp. 133–152, JB Lippincott: Philadelphia.

HENDERSON BW AND FINGAR VH. (1987). Relationship of tumour hypoxia and response to photodynamic treatment in an experimental mouse tumor. *Cancer Res.*, **47,** 3110–3114.

HENDERSON BW, WALDOW SM, MANG TS, POTTER WR, MALONE PB AND DOUGHERTY TJ. (1985). Tumour destruction and kinetics of tumour cell death in two experimental mouse tumors following photodynamic therapy. *Cancer Res.*, **45,** 572–576.

HIRSCH BD, WALZ NC, MEEKER BE, ARNFIELD MR, TULIP J, MCPHEE MS AND CHAPMAN JD. (1987). Photodynamic therapy-induced hypoxia in rat tumors and normal tissues. *Photochem. Photobiol.*, **46,** 847–852.

HODGKISS RJ, JONES G, LONG A, PARRICK J, SMITH KA, STRATFORD MRL AND WILSON GD. (1991). Flow cytometric evaluation of hypoxic cells in solid experimental tumours using fluorescence immunodetection. *Br. J. Cancer*, **63,** 119–125.

KOCH CJ. (1993). Unusual oxygen concentration dependence of toxicity of SR4233, a hypoxic cell toxin. *Cancer Res.*, **53,** 3992–3997.

MARSHALL RS AND RAUTH M. (1986). Modification of the cytotoxic activity of mitomycin c by oxygen and ascorbic acid in chinese hamster ovary cells and a repair-deficient mutant. *Cancer Res.*, **46,** 2709–2713.

MOORE RB, CHAPMAN JD, MERCER JR, MANNAN RZ, WIEBE LI, MCEWAN AJ AND MCPHEE MS. (1993). Measurement of PDT-induced hypoxia in Dunning prostate tumors by iodine-123 iodoazomycin arabinoside. *J. Nucl. Med.*, **34,** 405–411.

MULCAHY RT. (1984). Effect of oxygen on misonidazole chemo-sensitization and cytotoxicity in vitro. *Cancer Res.*, **44,** 4409–4413.

RIJKEN PFJW, BERNSEN HJJA AND VAN DER KOGEL AJ. (1995). Application of an image analysis system to the quantitation of tumor perfusion and vascularity in human glioma xenografts. *Microvascular Res.*, **50.**

STAR WM, MARIJNISSEN HPA, BERG-BLOK AE, VERSTEEG JAC, FRANKEN KAP AND REINHOLD HS. (1986). Destruction of rat mammary tumour and normal tissue microcirculation by hematoporphyrin derivative photoirradiation observed *in vivo* in sandwich observation chambers. *Cancer Res.*, **46,** 2532–2540.

TAYLOR YC AND RAUTH AM. (1982). Oxygen tension, cellular respiration, and redox state as variables influencing the cytotoxicity of the radiosensitizer misonidazole. *Radiat. Res.*, **91,** 104–123.

THOMLINSON RH AND GRAY LH. (1955). The histological structure of some human lung cancers and the possible implications for radiotherapy. *Br. J. Cancer*, **9,** 539–549.

TROTTER MJ, CHAPLIN DJ, DURAND RE AND OLIVE PL. (1989). The use of fluorescent probes to identify regions of transient perfusion in murine tumors. *Int. J. Radiat. Oncol. Biol. Phys.*, **16,** 931–934.

TROTTER MJ, OLIVE PL AND CHAPLIN DJ. (1990). Effect of vascular marker Hoechst 33342 on tumour perfusion and cardiovascular function in the mouse. *Br. J. Cancer*, **62,** 903–908.

TWENTYMAN PR, BROWN JM, GRAY JW, FRANKA AJ, SCOLES MA AND KALLMAN RF. (1980). A new mouse tumour model system (RIF-1) for comparison of end-point studies. *J. Natl Cancer Inst.*, **64,** 594–604.

VAUPEL P, KALLINOWSKI F AND OKUNIEFF P. (1989). Blood flow, oxygen and nutrient supply, and metabolic microenvironment of human tumors: a review. *Cancer Res.*, **49,** 6449–6463.

VAN GEEL IPJ, OPPELAAR H, OUSSOREN Y AND STEWART FA. (1994). Changes in perfusion of mouse tumours after photodynamic therapy. *Int. J. Cancer*, **56,** 224–228.

VAN GEEL IPJ, OPPELAAR H, OUSSOREN Y, SCHUITMAKER JJ AND STEWART FA. (1995*a*). Mechanisms for optimising PDT: second generation photosensitizers in combination with mitomycin C. *Br. J. Cancer*, **72,** 344–350.

VAN GEEL IPJ, OPPELAAR H, OUSSOREN Y AND STEWART FA. (1995*b*). Photosensitizing efficacy of mTHPC-PDT compared to Photofrin-PDT in the RIF-1 tumour and normal skin. *Int. J. Cancer*, **60,** 388–394.